# Altered Protein and Gene Expression of *Beclin-1* Correlates with Poor Prognosis of Hcv-Associated Hepatocellular Carcinoma in Egyptian Patients

**DOI:** 10.31557/APJCP.2021.22.4.1115

**Published:** 2021-04

**Authors:** Nermine Ahmed Ehsan, Asmaa M Mosbeh, Sally Waheed Elkhadry, Asmaa Ibrahim Gomaa, Maha Mohamed Elsabaawy, Dina Shehata Elazab

**Affiliations:** 1 *Department of Pathology, National Liver Institute, Menoufia University, Egypt. *; 2 *Molecular Pathology, National Liver Institute, Menoufia University, Egypt. *; 3 *Epidemiology and Preventive Medicine, National Liver Institute, Menoufia University, Egypt. *; 4 *National Liver Institute, Menoufia University, Shebin El-Kom, Egypt. *

**Keywords:** Beclin-1, Autophagy, HCC, HCV

## Abstract

Autophagy modulation has recently been addressed as a novel target for overcoming therapeutic resistance in hepatocellular carcinoma (HCC) to currently available anti-HCC therapy. The aim of this study was to investigate the protein and gene expression of *Beclin-1 *and its correlation with prognosis in HCV-associated HCC in Egyptian patients. This prospective study included 50 patients with HCV-associated-HCC, treated with surgical resection. Immunohistochemistry of antibody and quantitative real-time PCR of *Beclin-1* gene were assessed in liver tissues of HCC. A normal-like expression pattern of *Beclin-1* was found in 100% of adjacent liver tissues, while in HCC three various patterns were recognized: negative expression [18 (36%)], over expression [16 (32%)] and normal pattern [16 (32%)] (p=0.001). *Beclin-1 mRNA* in HCC tissues correlated with protein expression with correlation coefficient of 0.774 (p<0.001). Patients with negative expression of *Beclin-*1 had a significantly poor overall survival rates compared with patients with normal-like expression pattern (P<0.007), which was confirmed by multivariate analysis (p=0.01). Over-expression of *Beclin-1* was significantly associated with vascular invasion (P<0.003). However, high tumor histological grade, focal lesion multiplicity, presence of involved margin or cirrhosis were insignificantly related to *Becin-1*. *Beclin-1* altered expression has an important role in development and prognosis of HCC.

## Introduction

Hepatocellular carcinoma (HCC), a global health problem, is the second leading cause of cancer related mortality as reviewed by Gomaa and Waked (Gomaa et al., 2017). Despite advancement in diagnostic and therapeutic modalities, most HCC patients are diagnosed at an advanced tumor stage and are associated with poor prognosis (Kudo, 2017). Although several clinical, histological and molecular factors (Ferrín et al., 2015) have been reported to be associated with the prognosis of HCC, more effective biomarkers are necessary to predict the clinical outcome of patients with HCC (Scaggiante et al., 2014). Recently, a large number of genes that may have significant involvement in the process of hepatocarcinogenesis were identified (Levy and Thorburn, 2020). However, the mechanisms by which these factors may promote initiation or progression to HCC are not well understood.

Autophagy is a non-apoptotic form of programmed cell death as a catabolic response of cells to stress. This process allows cells to maintain homeostasis and recycling intracellular constituents. Autophagy may be upregulated in response to different types of stresses including hypoxia, nutrient and growth factor deprivation, endoplasmic reticulum (ER) stress, pathogen entry, or chemotherapeutic drugs (Udristioiu et al., 2019). Also, autophagy is a survival pathway required for cellular viability and facilitating cell survival under stressful conditions (Oral et al., 2016). However, stimulation of autophagy may lead to cell death through unknown mechanisms. An alternative non-apoptotic programmed cell death mechanism, “autophagic cell death” was proposed (Madrigal-Matute and Cuervo, 2016) .

Autophagy has been described as one of the central pathways for liver health and disease. In starved animals, a grand majority of total protein and glycogen degradation in the liver depends on autophagic degradation (Wang et al., 2019). On the other hand, autophagy is related to several liver diseases, including fatty liver disease and HCC (Akkoç and Gözüaçık, 2018).

Beclin-1, the mammalian orthologue of yeast Atg6/Vps30, is a coiled coil protein that act as a tumor suppressor (Vega-Rubín-de-Celis, 2020). Loss of Beclin-1 in mice was found to be correlated with a reduction in autophagic vacuole formation, and an unpredicted increase in human melanoma, colon, ovarian, brain cancers and HCC (Ávalos et al., 2014). It had been stated that beclin-1 is a haplo-insufficient tumor suppressor gene, which can promote cancer through impaired autophagy and increased cell proliferation (Abdou et al., 2020). Consequently, the present study was designed to determine the expression of *Beclin-1* in HCV associated-HCC cases and adjacent non-neoplastic hepatic tissues, along with its correlation to demographic, clinicopathological factors, and patient’s outcome regarding overall survival and recurrence.

## Materials and Methods

This prospective study comprised 50 patients who were diagnosed with HCC and had undergone curative resection in a tertiary referral center between January 2013 and December 2014. The included patients had primary HCC on top of chronic hepatitis C virus (HCV) infection with no history of previous anti-cancer treatment. 

Diagnosis of HCC was confirmed by multislice triphasic computed tomography (CT) scan with or without elevated alpha fetoprotein more than 200 ng/ml (Abdou et al., 2020).

*Compliance with Ethical Standards*

Informed consent was obtained from every participant in this study after explaining clearly the purpose of the study. Data of patients collected from medical records were dealt with confidentially and anonymously. This study was approved by the Ethics Committee of The National Liver Institute, Menoufia University and was conducted in accordance with the Declaration of Helsinki,1964. 

The patients had follow-up that ranged from 13 to 72 months with a median of 58.4 months. The follow-up period was defined as the duration from the date of operation to the date of last follow-up or death. Deaths from other causes were treated as censored cases. Overall survival (OS) was evaluated by the duration between the dates of surgery and death. 

For all patients, demographic data, clinical and pathological records were reviewed. Tumor size, liver cirrhosis, serum alpha fetoprotein (AFP) values and metastasis were reported. 

*Immunohistochemical (IHC) staining*

Tissue microarrays (TMA) were constructed for tumorous and adjacent non tumorous tissue (ANT) for all studied cases (Abdou et al., 2020). Each sample tissue was represented in three different sites on the TMA block. Four-micron thick sections were cut from all TMA blocks prepared. IHC was carried out for all tumor and adjacent non tumor tissues. After deparaffinization and rehydration, heat-induced antigen retrieval was performed using the DAKO high pH antigen retrieval solution in a vegetable steamer for 20 minutes at 97°C followed by incubation for an additional 20 minutes in the warm buffer. Anti-Beclin-1 rabbit polyclonal antibody (ab62557- Abcam, Cambridge, UK) was incubated overnight at 4C° with a 1:100 dilution. Detection of the immunostaining was carried out utilizing the EnvisionTM FLEX/HRP detection system (DAKO A/S, Glostrup, Denmark) with the 3-diaminobenzidine (DAKO) as chromogen and counterstained with hematoxylin. Negative control was conducted for each IHC run by replacing the primary antibody by TBS (Tris buffer saline).

*Interpretation of IHC results*

Stained sections were evaluated by two independent histopathologists without prior knowledge of the clinical information using an immunoreactive score (IRS). Scores were assigned for the intensity and percentage of positive staining of the cytoplasm in hepatocytes. Briefly, the IRS assigns subscores for percentage (0–4) and intensity (0–3) and multiplies the subscores to yield the IRS score, which ranged from 0-12. The percent positivity was scored as “0” (<5%), “1” (5–25%), “2” (>25–50%), “3” (>50–75%) or “4” (>75%). The staining intensity was scored as “0” (no staining), “1” (weakly stained), “2” (moderately stained) and “3” (heavy stained). In cases where differences in intensity were observed among the triplicates, the highest score was to be the final score. For statistical analysis, data were categorized into 3 groups. Negative expression group was assigned a score 0, low expression was assigned a score of 1- <6 and high expression was assigned to scores ≥6 (Qiu et al., 2014).

*Quantitative detection of mRNA encoding for Beclin-1 by Reverse Transcription PCR (qRT-PCR)*

RNA from frozen HCC tissue samples was isolated using the Trizol (Invitrogen, Carlsbad, CA) according to the manufacturer’s protocol. The extracted RNA was stored at -80˚C until the time of experiments. Complementary DNA (cDNA) was synthesized using the SuperScript VILO cDNA Synthesis Kit according to the manufacturer’s protocol (Invitrogen, Carlsbad, CA). Quantitative real-time PCR assay was carried out using beclin probe Hs00186838 (Lot: 1486524), normalized to PP1A control mix (Applied Biosystems Lot:1406019, Foster City, CA). The reactions were carried out in duplicate and performed on the VIIA 7. The relative expression levels and fold changes of the target gene expression, normalized to PP1A levels, were determined by the comparative CT method using the formula 2^-∆∆Ct^. The relative gene expression is usually set to 1 for reference sample because ∆∆Ct is equal to 0 and therefore 20 is equal to 1 (Obada et al., 2017).

*Statistical analysis*

Statistical analysis was conducted with SPSS software, version 22.1 (SPSS, Inc., Chicago, IL, USA) and a P value <0.05 was considered significant. Categorical variables were analyzed using the *χ*^2^ contingency test and the exact probability test. Differences between HCC tissues and adjacent non-tumor tissues were tested for significance using Wilcoxon signed rank tests. The Spearman’s rank correlation test was utilized to reveal the correlation between *Beclin-1* immuno-expression with pathological characteristics and AFP level. Univariate survival analyses were used to examine prognostic significance of* Beclin-1 *expression. Curves for overall survival (OS) were drawn according to the Kaplan–Meier method and difference was analyzed by log-rank test. In accordance with results from Cox univariate regression analyses, significant factors were evaluated by multivariate regression analyses to determine independent prognostic factors. 

## Results

*Expression of Beclin-1 in HCC and adjacent non tumor liver tissues by IHC*

**Figure 1:**
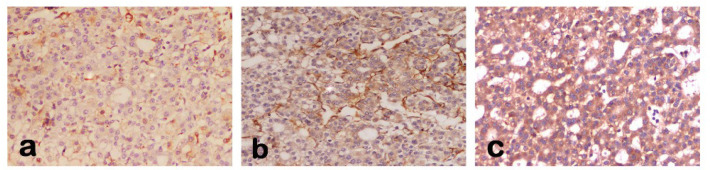
Immunostaining of Beclin-1 Antibody in Liver Tissues from HCC Cases Demonstrating the Three Various Patterns of *Beclin-1* Expression, a, negative; b, normal-like; c, over expression. Original mag.x200

IHC staining of Beclin-1 antibody revealed brownish staining of hepatocytes’ cytoplasm. All examined adjacent non tumor liver tissue revealed low expression of *Beclin-1* (IRS <6) corresponded to the normal expression pattern. IHC staining of Beclin-1 on HCC tissue revealed three various patterns of expression as shown in Figure 1. The patterns observed were: negative, normal-like expression (as in non-tumorous tissue, IRS <6) and over expression (IRS ≥6). The number of cases with various patterns of *Beclin-1* expression are represented in Table 1. A significant difference was observed between HCC and ANT samples (*χ*^2^ = 8.240, P = 0.016). 

*Expression of Beclin-1 mRNA in HCC tissues*

**Figure 2:**
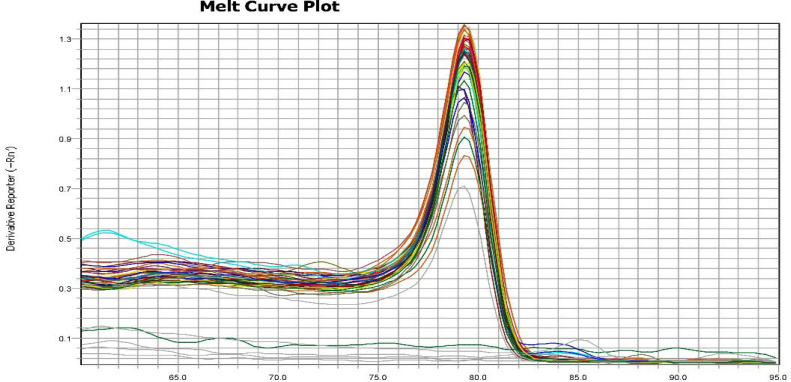
Melting Temperature Curve Plot of *Beclin-1* mRNA in HCC Tissues

All samples were specific to the Beclin-1 primer and exhibited the same melting temperature as shown in Figure 2. The final result was presented as fold change of target gene expression, *Beclin-1*, relative to a reference sample normalized to a reference gene. Three different patterns appeared in PCR quantitation of *Beclin-1 mRNA*. Normal expression pattern (17 out of 50) revealed as the reference genes. Negative expression pattern (18 out of 50) ranged from 0.1 to 0.6 folds. Over expression pattern (15 out of 50) ranged from 2.4 to 26 folds. The expression of Beclin-1 protein by IHC was highly correlated with Beclin-1 mRNA in HCC tissues with correlation coefficient of 0.774 (p<0.001). 

*Association of Beclin-1 expression with clinicopathologic features of HCC*

Table 2 demonstrated the relation between *Beclin-1* expression and demographic, laboratory and pathological features in HCC cases. There was no significant correlation between *Beclin-1* expression with either age, gender or AFP levels. *Beclin-1* over-expression in HCC was positively correlated with vascular invasion (P =0.058). Vascular invasion was detected in 33 out of 50 HCC cases. Over expression of *Beclin-1* was associated with 78.7% of vascular invasion. There were no significant associations between *Beclin-1* expression and the presence of liver cirrhosis. *Beclin-1* negative or over expression was negatively correlated with HCC Edmondson grade although did not reach statistical significance (P =0.10). *Beclin-1* expression was stronger in HCC with Edmondson I–II grade (73.5%, 25/34) than HCC with III–IV grade (26.5%, 9/34). Similarly, *Beclin-1* negative or over expression was insignificantly correlated with focal lesion or involved margin. Multinodular HCC revealed 70.6% (24/34).* Beclin-1* negative and over expression was associated with free margin (67.6%, 23/24) than in HCC with involved capsule (32.4%, 11/34). 

*The association of Beclin-1 expression with patients’ survival*

**Figure 3:**
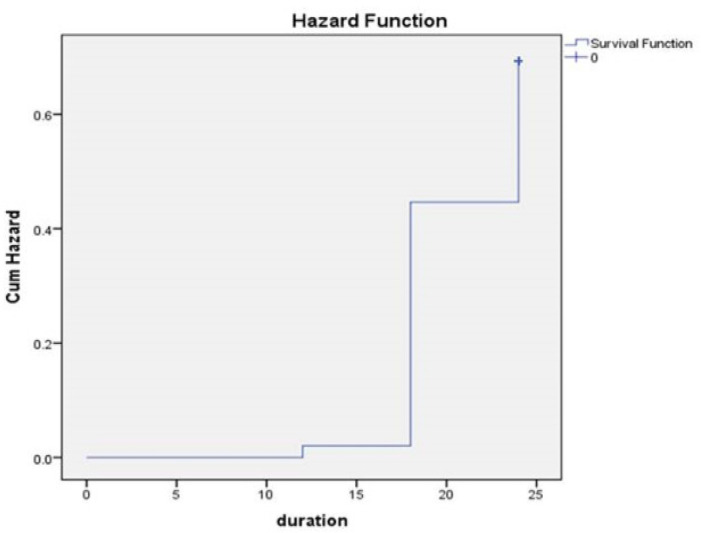
Kaplan Meier Curve of Overall Survival in HCC Cases, duration in Months

Kaplan-Meier analysis of patient survival revealed that negative expression of *Beclin-1* may point to a poor prognosis for HCC patients. The mean OS of patients for *Beclin-1* with negative expression, normal pattern and over expression was 20.3, 23.7 and 18.4 months, respectively, with the log-rank test demonstrating significant difference (P = 0.037, Figure 3). 

The prognosis of the *Beclin-1* normal pattern expression group was better than that of the over expression and the negative expression groups (P= 0.001 and 0.009 respectively). Although no significant difference in OS was observed between *Beclin-1 *over expression or negative expression groups (P = 0.48). *Beclin-1* negative expression was significantly associated with poor OS by both univariate (0.007), and multivariate Cox regression analysis (0.012).

Cox regression analysis was used to compare *Beclin-1* expression with clinicopathological features of survival prediction as shown in Table 3. Using univariate Cox regression analysis, vascular invasion and multinodular tumor were significantly associated with poor OS (P=0.004, 0.03 respectively). HCC recurrence was found to be not significantly related to any of demographic, pathological features, or *Beclin-1* negative expression on either univariate or multivariate analysis as shown in Table 4.

**Table 1:** *Beclin-1* Protein Expression in HCC and ANT Tissues

Sample	Number of Cases	*Beclin-1* expression			χ^2^/P
		(−, under expression) %	(+, normal expression) %	(++, over expression) %	
HCC	50	18 (36%)	16 (32%)	16 (32%)	0.001
ANT	50	0 (0%)	50 (100%)	0 (0%)	

HCC, hepatocellular carcinoma; ANT, adjacent non-tumorous tissue; P value <0.05 is considered significant, more than 0.001 is highly significant, while more 0.05 is insignificant.

**Table 2:** *Beclin-1* Expression Relation to Demographic, Laboratory, and Tumor Pathological Features

Characteristics	N	*Beclin-1 *expression			X^2^ /R# (P)
	Total	(−) %	(+) %	(++) %	
Age					
<60 y	22 (44%)	6 (33.3%)	8 (50%)	8 (50%)	0.522
≥60 y	28 (56%)	12 (66.7%)	8 (50%)	8 (50%)	
Sex					
Male	40 (80%)	16 (88.9%)	14 (87.5%)	10 (62.5%)	0.105
Female	10 (20%)	2 (11.1%)	2 (12.5%)	6 (37.5%)	
AFP					0.138
<50 ng/ml	44 (88%)	14 (77.8%)	14 (87.5%)	16 (100%)	
≥50 ng/ml	6 (12%)	4 (22.2%)	2 (12.5%)	0 (0%)	
Liver cirrhosis					
No	7 (14%)	1 (5.6%)	2 (12.5%)	4 (25%)	0.259
Yes	43 (86%)	17 (94.4%)	14 (87.5%)	12 (75%)	
Tumor size					
≤2 cm	2 (4%)	1 (5.6%)	1 (6.2%)	0 (0%)	0.829
2 cm-10 cm	42 (84%)	14 (77.8%)	13 (81.2%)	15 (93.8%)	
>10 cm	6 (12%)	3 (16.7%)	2 (12.5%)	1 (6.2%)	
Edmondson grades					
I-II	33 (66%)	11 (61.1%)	8 (50%)	14 (87.5%)	0.07
III-IV	17 (34%)	7 (38.9%)	8 (50%)	2 (12.5%)	
Capsule integrity					
free	34 (68%)	13 (72.2%)	11 (68.8%)	10 (62.5%)	0.829
Involved	16 (32%)	5 (27.8%)	5 (31.2%)	6 (37.5%)	
Vascular invasion					
No	20	6 (30)	11 (55)	3 (15)	11.501
Yes	30	7 (23.3)	5 (16.7)	18 (60)	(0.003)*
Tumor number					
Single	34 (68%)	12 (66.7%)	10 (62.5%)	12 (75%)	0.74
Multiple	16 (32%)	6 (33.3%)	6 (37.5%)	4 (25%)	

HCC, hepatocellular carcinoma; ANT, adjacent non-tumorous tissue; P value <0.05 is considered significant, more than 0.001 is highly significant, while more 0.05 is insignificant.

**Table 3:** Univariate and Multivariate Cox Regression Analysis for Predictors of Overall Survival

Variables	Univariate analysis Hazard ratio (95% CI)	P	Multivariate analysis Hazard ratio (95% CI)	
Age	0.995 (0.955-1.037)	0.819	0.983 (0.030-1.029)	0.458
Sex (male vs. female)	0.788 (0.315-1.973)	0.611	0.1.36 (0.501-3.72)	0.543
Tumor size ( ≤ 5 cm vs. >5 cm )	0.959 (0.438-2.102)	0.917	1.319 (0.511-3.401)	0.567
Edmondson grades (I+II vs. III+IV)	2.287 (0.857-6.101)	0.098	2. 705 (0.924-7.919)	0.069
Capsular infiltration (Negative vs. Positive)	0.642 (0.288-1.429)	0.277	0.641 (0.262-1.269)	0.33
Vascular invasion (Positive vs. Negative)	4.599 (1.607-13.16)	0.004	3.301 (1.018-10.7)	0.047
Cirrhosis background	0.682 (0.234-1.991)	0.484	2.122 (0.659-6.834)	0.207
Multinodular tumor (Multiple vs. Single)	2.573 (1.087-6.087)	0.032	1.565 (0.634-3.863)	0.331
*Beclin-1* (over expression or normal vs. low expression)	0.329 (0.147-0.738)	0.007	0.311 (0.126-0.771)	0.012

HCC, hepatocellular carcinoma; ANT, adjacent non-tumorous tissue; P value <0.05 is considered significant, more than 0.001 is highly significant, while more 0.05 is insignificant.

**Table 4:** Factors Affecting HCC Recurrence at 6 Months

Variables	Univariate analysis		Multivariate analysis	
	Hazard ratio (95% CI)	P	Adjusted hazard ratio (95% CI)	P
Age	1.006 (0.965-1.049)	0.764	0.994 (0.947-1.043)	0.806
Sex (male vs. female)	0.821 (0.33-2.04)	0.672	2.135 (0.742-6.130)	0.159
Tumor size ( ≤ 5 cm vs. >5 cm )	1.281 (0.592-2.77)	0.53	1.064 (0.441-2.563)	0.891
Edmondson grades (I+II vs. III+IV)	1.030 (0.459-2.31)	0.943	0.912 (0.349-2.385)	0.851
Capsular infiltration (Negative vs. Positive)	1.344 (0.609-2.964)	0.464	1.071 (0.440-2.605)	0.88
Vascular invasion (Positive vs. Negative)	2.036 (0.851-4.876)	0.11	2.197 (0.370-6.616)	0.162
Cirrhosis background	0.887 (0.306-2.576)	0.826	0.643 (0.198-2.087)	0.462
Multinodular tumor (Multiple vs. Single)	1.120 (0.499-2.513)	0.783	1.065 (0.410-2.767)	0.898
*Beclin-1* (over expression or normal vs. negative expression)	0.495 (0.228-1.078)	0.077	0.506 (0.219-1.170)	0.111

Adjusted for gender, grade, nodularity, vascular invasion and *Beclin-1* expression, HCC, hepatocellular carcinoma; ANT, adjacent non-tumorous tissue; P value <0.05 is considered significant, more than 0.001 is highly significant, while more 0.05 is insignificant.

## Discussion

The defined role of autophagy in genomic stability through the precancerous phase might be completely ameliorated in the stage of cancer occurrence (Levy et al., 2017). Promotion of tumor growth, along with degradation of deformed organelles were of importance in specifying the attitude of autophagy in the cancerous phase (Zhai et al., 2013).

The need for more sensitive molecular predictors of survival in HCC cases had been challenged in many studies with unsatisfactory impacts. The autophagy gene, Beclin-1, had been studied in stomach, ovaries, esophagus, and colon cancers (Hu et al., 2020). Over-expression of *Beclin-1* had been demonstrated in pancreatic and nasopharyngeal cancer (Hu et al., 2016). However, the role of *Beclin-1* expression in HCC as a prognostic marker affecting survival is yet unrevealed. The reported altered expression (either negative or over expression) of the proapoptotic *Beclin-1* in HCC had added more uncertainty about its oncogenic capabilities (Liang et al., 2018). 

In the current study, normal pattern expression of *Beclin-1* was detected in all adjacent non tumorous tissue (100%), with negative expression in 18/50 (36%) and over-expression in 16/50 (32%) of investigated HCC cases. This finding has been observed in cancer where both tumor promoting and tumor inhibiting factors of autophagy coexist and are thought to be of great importance (Koukourakis et al., 2010). 

Qiu et al., (2014), had supported these results reporting that *Beclin-1 *was over expressed in HCC significantly than the normal pattern of expression observed in normal and cirrhotic tissues. Similarly, were the results of Ying-Hong et al., (2009) who reported Beclin-1 over expression in 31.7% of HCC cases. Also, Al-Shenawy (2016), had found that* Beclin-1* revealed negative expression in HCC cases while over-expressed in liver tissues of chronic viral hepatitis. Consequently, a suggested role of autophagy in chronic hepatitis progression rather than HCC occurrence which was mainly linked to the suppressor function of Beclin-1 (Yun et al., 2018). 

The point at which *Beclin-1 *expression started to be altered is the most critical landmark pointing to HCC development. Definite recognition of this point could be of value in HCC prevention. It has been well established in the literature that autophagy plays a dual role in cancer. Autophagy can clear oncogenic protein subtypes and toxic unfolded proteins leading to suppression of tumor formation. On the other hand, established tumors revealed increased autophagic flux that promote tumor cells to survive and grow (Xiaohua et al., 2020). 

In the current study, over expression of *Beclin-1* in HCC cases could not be ignored and should emphasize a gloss that this gene alterations must be seriously considered. However further research should be reproduced for better delineation of this paradigm.

The reported increase Beclin-1 in chronic hepatitis as documented by Al-Shenawy (2016), could be a body defense mechanism to prevent the progression towards carcinogenesis. Whereas, negative expression of *Beclin-1*, being a tumor suppressor gene, favors the development of hepatocarcinogenesis.

This study had uniquely demonstrated that expression of Beclin-1 protein was highly correlated with Beclin-1 mRNA in HCC tissues with correlation coefficient of 0.774 (p<0.001). 

In the current study, there were no significant associations between *Beclin-1* expression and age, gender or AFP. Many studies had also negated any relevance of Beclin-1 to the impacts or progression of HCC on general health (Lee et al., 2013; Osman et al., 2015; Wu et al., 2017).

Regarding pathological features of HCC, *Beclin-1 *expression was stronger in HCC with Edmondson I–II grade than HCC with III–IV grade. All HCC studied cases with vascular invasion and multinodular tumor revealed altered *Beclin-1 *expression either negative or over expression. These results were backing the same concept of linking HCC to negative *Beclin-1* expression (Al-Shenawy et al., 2016). On the contrary were the results of Qiu et al., (2014), who reported a positive correlation between *Beclin-1* expression in HCC with liver cirrhosis, results from this study demonstrated no significant correlation between liver cirrhosis with *Beclin-1* expression. This could be attributed to different underlying etiology of HCC in their study population than our unique HCV-genotype 4 associated HCC. 

The average HCC median survival was found to be 22.8 months in an Egyptian review (Gomma et al., 2017). Waziry et al., (2018), in their study, had demonstrated factors associated with poorer survival in adjusted analyses as: INR (HR = 1.81, p = 0.01), alpha-fetoprotein (AFP) ≥200 (HR = 1.41, p = 0.02), higher CTP score (HR = 2.48, p < 0.01), and advanced BCLC stage (HR = 1.85, p < 0.01). The debatable linkage between negative expression of *Beclin-1* to poor survival was questioned in many studies. While Qui et al., (2014), had approved this linkage, Wu et al, had completely denied it. A recent meta-analysis had suggested that *Beclin-1* over expression might grantee a better 5-year survival in HCC cases (Liang et al., 2018). 

In the current study, the median overall survival (OS) revealed significant correlation of negative expression of *Beclin-1* and poor prognosis for HCC patients. *Beclin-1 *over-expression was insignificantly associated with poor OS either by univariate or multivariate analysis.

All these suggested data might prevail the way of considering *Beclin-1* altered expression as a sign of HCC development and tumor aggression. Negative expression of *Beclin-1* may reflect a poor impact on OS. These suggestions are logically accepted for the fact that most HCC cases died of complications of liver cirrhosis even before the lethal effects of HCC are due. Considering the eventual molecular print of *Beclin-1 *negative expression in HCC occurrence, a close monitoring of this altered gene in cases of liver cirrhosis might be of significance in HCC early detection. 

In conclusion, through the Beclin-1 paradigm we have learned how genetic alterations could modulate hepatocarcinogenesis. Beclin-1 as a marker of autophagy has an important role in HCC development and progression. Negative expression of *Beclin-1* gene may determine aggressive tumor behavior reflected by the overall survival. 

## Author Contribution Statement

Nermine Ehsan: study concept and design, interpretation of IHC results, contributed to data acquisition, wrote the manuscript. Asmaa Mosbeh: conducted quantitative detection of mRNA encoded for *Beclin-1* gene by qRT-PCR, preparing of figures. Sally Waheed: performed statistical analysis. Asmaa Gomaa and Maha Elsabaawy: involved in patients’ recruitment, clinical and follow up and contributed to data acquisition, draft the manuscript. Dina Elazab: interpretation of IHC, literature review, data acquisition. All authors revised the manuscript.

## References

[B1] Abdou AG, Holah N, Elazab DS (2020). Hepatocellular carcinoma score and subclassification into aggressive subtypes using immunohistochemical expression of p53, ß-Catenin, CD133 and Ki67. Appl Immunohistochem Mol Morphol.

[B2] Akkoç Y, Gözüaçık D (2018). Autophagy and liver cancer. Turk J Gastroenterol.

[B3] Al-Shenawy HA-S (2016). Expression of Beclin-1, an autophagy-related marker, in chronic hepatitis and hepatocellular carcinoma and its relation with apoptotic markers. APMIS.

[B4] Ávalos Y, Canales J, Bravo-Sagua R (2017). Tumor suppression and promotion by autophagy. BioMed Res Int.

[B5] Castelli G, Pelosi E, Testa U (2017). Liver cancer: Molecular characterization, clonal evolution and cancer stem cells. Cancers (Basel).

[B6] Ferrín G, Aguilar-Melero P, Rodríguez-Perálvarez M (2015). Biomarkers for hepatocellular carcinoma: diagnostic and therapeutic utility. Hepat Med.

[B7] Gomaa A, Waked I (2017). Management of advanced hepatocellular carcinoma: review of current and potential therapies. Hepatoma Res.

[B8] Hu YJ, Zhong JT, Gong L (2020). Autophagy-related Beclin 1 and head and neck cancers. Onco Targets Ther.

[B9] Hu Z, Zhong Z, Huang S (2016). Decreased expression of Beclin 1 is significantly associated with a poor prognosis in oral tongue squamous cell carcinoma. Mol Med Rep.

[B10] Koukourakis MI, Giatromanolaki A, Sivridis E (2010). Beclin 1 over- and underexpression in colorectal cancer: distinct patterns relate to prognosis and tumour hypoxia. Br J Cancer.

[B11] Kudo M (2017). A new era of systemic therapy for hepatocellular carcinoma with regorafenib and lenvatinib. Liver Cancer.

[B12] Lee YJ, Hah YJ, Kang YN (2013). The autophagy-related marker LC3 can predict prognosis in human hepatocellular carcinoma. PLoS One.

[B13] Levy JMM, Thorburn A (2020). Autophaghy in cancer: moving from understanding mechanisms to improving therapy response in patients. Cell Death Differ.

[B14] Levy JMM, Towers CG, Thorburn A (2017). Targeting autophagy in cancer. Nat Rev Cancer.

[B15] Liang C, Li W, Ge H (2018). Role of Beclin1 expression in patients with hepatocellular carcinoma: a meta-analysis. Onco Targets Ther.

[B16] Madrigal-Matute J, Cuervo M (2016). Regulation of liver metabolism by autophagy. Gastroenterology.

[B17] Obada M, El-Fert A, Hashim M (2017). Impact of genetic polymorphisms of four cytokine genes on treatment induced viral clearance in HCV infected Egyptian patients. EJMHG.

[B18] Oral O, Akkoc Y, Bayraktar O (2016). Physiological and pathological significance of the molecular cross-talk between autophagy and apoptosis. Histol Histopathol.

[B19] Osman NA, Abd El-Rehim DM, Kamal IM (2015). Defective beclin-1 and elevated hypoxia-inducible factor (HIF)-1α expression are closely linked to tumorigenesis, differentiation, and progression of hepatocellular carcinoma. Tumour Biol.

[B20] Scaggiante B, Kazemi M, Pozzato G (2014). Novel hepatocellular carcinoma molecules with prognostic and therapeutic potentials. World J Gastroenterol.

[B21] Qiu DM, Wang GL, Chen L (2014). The expression of beclin-1, an autophagic gene, in hepatocellular carcinoma associated with clinical pathological and prognostic significance. BMC Cancer.

[B22] Udristioiu A, Nica-Badea D (2019). Autophagy dysfunctions associated with cancer cells and their therapeutic implications. Biomed Pharmacother.

[B23] Vega-Rubín-de-Celis S (2020). The role of Beclin 1-dependent autophagy in cancer biology. Cancer Biol.

[B24] Wang H, Liu Y, Wang D (2019). The upstream pathway of mTOR-mediated autophagy in liver diseases cells. Cell.

[B25] Waziry R, Gomaa A, Waked I (2018). Determinants of survival following hepatocellular carcinoma in Egyptian patients with untreated chronic HCV infection in the pre-DAA era. Arab J Gastroenterol.

[B26] Wu DH, Jia CC, Chen J (2014). Autophagic LC3B overexpression correlates with malignant progression and predicts a poor prognosis in hepatocellular carcinoma. Tumour Biol.

[B27] Xiaohua L, He S, Ma B (2020). Autophagy and autophagy-related proteins in cancer. Mol Cancer.

[B28] Ying-Hong S, Zhen-Bin D, Jian Z (2009). Prognostic significance of Beclin 1-dependent apoptotic activity in hepatocellular carcinoma. Autophagy.

[B29] Yun CW, Lee SH (2018). The role of autophagy in cancer. Intl J Mol Sci.

[B30] Zhai H, Song B, Xu X (2013). Inhibition of autophagy and tumor growth in colon cancer by miR-502. Oncogene.

